# Use of Powered Prosthesis for Children with Upper Limb Deficiency at Hyogo Rehabilitation Center

**DOI:** 10.1371/journal.pone.0131746

**Published:** 2015-06-30

**Authors:** Mitsunori Toda, Takaaki Chin, Yaeko Shibata, Futoshi Mizobe

**Affiliations:** 1 Department of Orthopaedic Surgery, Hyogo Rehabilitation Center, Kobe, Japan; 2 Department of Physical Medicine and Rehabilitation, Hyogo Rehabilitation Center, Kobe, Japan; 3 Department of Rehabilitation Science, Kobe University Graduate School of Medicine in Hyogo Rehabilitation Center, Kobe, Japan; University of Sheffield, UNITED KINGDOM

## Abstract

**Background:**

There has been no research investigating the use of powered prosthetic for children in Japan.

**Objective:**

To gain better insight into the state of powered prosthesis usage and identify a ratio of rejection among children.

**Methods:**

Subjects were 37 unilateral below elbow amputees between the ages of 0 and 16 at the time of their first experienced fitting with a powered prosthesis at our Center. The information was collected from medical records and through face-to-face interviews, and we examined rejection rate and the factors affecting the use of powered prosthesis.

**Results:**

The rate of discontinuation was 21.6% as 8 of the 37 children stopped using powered prosthesis. All of them were fitted their prosthesis after 2 years of age, and they rejected prosthesis between 5 to 19 years. We found that the level of amputation had no influence on the use of a powered prosthesis.

**Conclusions:**

Children fitted before 2 years of age tend to accept their powered prosthesis than those fitted after 2 years. Multidisciprinary team approach, adequate rehabilitation, detailed follow-up and involvement of parents are quite important for introducing powered prosthesis for children.

## Introduction

In the 1980’s research papers were issued one after another reporting on the effectiveness of the application of powered prosthesis for young children in Canada and Sweden [1,2,3]. Progress in prosthetic limb technology has enabled the miniaturization of powered prostheses, leading to wider use by children with upper limb deficiency in the U.S., Europe, and other regions. On the other hand, in Japan, though there were some examples of powered prosthesis being introduced on a trial basis for use with children, these practices were unfortunately abandoned. Since then there has been no efforts to reconsider the introduction of powered prosthesis for children, which is why wider use has not been observed among Japanese children.

Generally speaking, children with congenital upper limb deficiency are able to carry out necessary daily activities with their remaining body functions even without using a prosthesis [4,5]. Nonetheless, the use of a powered prosthesis has some advantages, one of which is enhanced bimanual activities [5,6,7]. This leads to the possibility that various activities a child has given up because they are difficult to perform with a single hand can be fully accomplished with the use of prosthesis. However, as children grow up, their lifestyles also change and there are greater demands to perform not only simple tasks, but also complicated ones. As a consequence, children become more aware of the limits of a powered prosthesis and tend to rely on it less [4,8]. In addition, such children have to face various mental and social problems related to upper limb deficiency during their formative years [8,9]. Therefore, it is not easy for a child to continually use a powered prosthesis from early childhood to early adolescence. To address this issue, we established a framework in 2002 (the first in Japan) to allow children to undergo training for the fitting a powered prosthesis at any time through daily clinical care. In the past, there has been no follow-up study to investigate the use of powered prosthetic among children in Japan. This is the first investigation that addresses this issue in Japan. The aim of this research was to obtain a clearer understanding of the state of powered prosthesis usage, identify a ratio of rejection among children who began training in the use of powered prosthesis at Hyogo Rehabilitation Center, and examine factors that have an influence on the use of the powered prosthesis.

## Patients and Methods

This research involved 37 children with congenital or acquired unilateral below elbow amputees who were under the age of 16 the first time they had an experienced fitting with a powered prosthesis at Hyogo Rehabilitation Center. All subjects showed regular development of motor function. Patients with above elbow amputations, bilateral amputations, additional handicaps and mental retardations were not included in this study. And we did not introduce the powered prosthesis if the family settings couldn’t support the child in training and using the powered prosthesis. The social status of all the parents was middle to upper middle, and they are cooperative with training and using powered prosthesis for their children. The children and their parents were informed of the purpose of this study, and verbal and written consent was obtained, and patient anonymity was preserved. This research was approved by the institutional review board of Hyogo Rehabilitation Center, and in accordance with protocol and followed the ethical and humane principles of research.

At the first consultation, doctors examined a patient, and initial introduction of powered prosthesis were given to the patients and their family by occupational therapists at our hospital. All the training were given at outpatient department, and a training program and maintenance of the prosthesis for the child and parents were instructed by the multidisciplinary team including doctors, occupational therapists, prosthetists, and engineers. All the children were seen every 3 months at our hospital. The follow-up period ranged from 2 to 12 years.

The details of the myoelectric prosthesis were almost identical for all patients excluding 2 cases that used switch operated prosthesis. Electric Hand 2000 (Ottobock, Duderstadt, Germany) was used. The Muenster socket, or the socket with window for the children with lower level of amputations or ulnar ray deficiencies was individually manufactured by the skilled prosthetists. A single electrode control was used for children fitted before the age of 3 years, and a double electrode control for fitted after 3 years. Children were given training sessions with MyoBoy (Ottobock) by occupational therapists at the authors hospital.

Information was collected regarding ages and when children started to use their powered prosthesis, the state of continued powered prosthesis usage, principal places (home, school, particular social situation) where a powered prosthesis was used, reasons for discontinuing the use of their powered prosthesis and the rate of discontinuation. Related information was collected from medical records and through face-to-face interviews. When interviewing a younger child, we asked a parent to be present to assist the child in answering these questions.

We divided the children into two groups: those who have continued using a powered prosthesis (user group) and those who discontinued using it (non-user group), and then examined whether age at the time of their first fitting, the level of amputation, and other several factors had an influence on their use of a powered prosthesis. The statistical significance of the differences was evaluated by use of Student’s t-test and Fisher's exact probability test. Results were considered significant at p<0.05.

## Results

Table 1 shows the characteristics of subjects in this study. They started to use their powered prosthesis when they were between the ages of 0 (10 months) and 16 years old, mean at 3.2 (median at 2.0) years old. Thirty-two of the children had congenital limb deficiencies, and 5 had acquired limb amputation. Among acquired amputees, 2 had sustained their amputation as the result of a trauma, 2 for malignancy, and 1 for ischemic necrosis during NICU care.

**Table 1:** Characteristics of subjects in this study.

Characteristic					
Age at fitting (y)	Range		Median		Mean±SD
	0–16		2.0		3.2±4.1
Sex	Male		Female		
	16		21		
Affected side	Right		Left		
	14		23		
Level of amputation	TR	WD	TC	UR	
	15	7	13	2	
Cause of amputation	Congenital		Acquired		
	32		5		

y: years, SD: standard deviation, TR: tranradial, WD: wrist disarticulation, TC: transcarpal, UR: ulnar ray

Table 2 shows the characteristics of users and non-users. There were 29 children in the users group, and 8 children in the non-users group. The rate of discontinuation was 21.6% (8/37). The ages at which the 8 children, who stopped using their powered prosthesis, had initially fitted their prosthesis from 4 to 16 (mean 10.6, median 9.5) years old, and when they discontinued usage were from 5 to 19 (mean 12.3, median 13.0) years old, respectively (Table 2). The age of non-users were significantly older compared with users at the time of their first fitting (p<0.05; Table 2). On the reason for rejection, seven of these children discontinued usage because they found it unnecessary. The other child discontinued usage because a sustainable follow-up program was impossible due to the long distance between home and our hospital. The level of amputation in non-users were 4 transradial amputations (rate of discontinuation; 26.7%), 2 wrist disarticulations (28.6%), and 2 transcarpal amputations (15.3%), respectively (Table 2). The influence of the level of amputation on the use of a powered prosthesis was judged through a comparison of the transradial amputation cases with wrist disarticulation and transcarpal amputation cases. In these cases, we found that the level of amputation had no influence on the use of a powered prosthesis. Likewise, sex and affected side had no influence on the use of the prosthesis.

**Table 2:** Charactristics of users and non-users and age when discontinuing the prosthesis.

Characteristic	Users(n = 29)				Non-users(n = 8)			
Age at fitting (y)	Range (Med)		Mean±SD		Range (Med)		Mean±SD	
	0–6(2.0)		2.4±1.8*		4–16(9.5)		10.6±4.2*	
Sex	M		F		M		F	
	13		16		3		5	
Affected side	R		L		R		L	
	12		17		2		6	
Level of amputation	TR	WD	TC	UR	TR	WD	TC	UR
	11	5	11	2	4	2	2	0
Age at discontinuation (y)	Range (Med)		Mean±SD		Range (Med)		Mean±SD	
	N/A		N/A		5–19(13.0)		12.3±4.4	
Rate of discontinuation	N/A				21.6%			

y: years, Med: Median, SD: standard deviation, TR: tranradial, WD: wrist disarticulation, TC: transcarpal, UR: ulnar ray, N/A: not applicable

*: p<0.05

As to the age of their first fitting with a powered prosthesis, 25 children were initially fitted with a powered prosthesis after the age of two years, and other 12 children before two years (Table 3). All the 8 children who stopped using their powered prosthesis were fitted after two years old. The rate of discontinuation was 32.0% (8/25) in fitted after two years old cases, and was higher compared with those fitted before two years old (Table 3). As to the situation for usage, 15 out of the 29 children were using a powered prosthesis not only at home, but also at school and at particular social situations. The remaining 14 children used it initially both at home and at their schools, but eventually came to use it only at home. This is because they could not obtain enough cooperation from the school, or occasionally they suffered from careless and hurtful comments about their disabilities and use of the prosthesis. Regarding the use of a powered prosthesis in particular social situations, two children used it while playing the violin and one used it while practicing flower arrangement. We found no relationship between the age of first fitting and the situation for usage (Table 3).

**Table 3:** Characteristics of subjects fitted before and after 2 years old, and the situation for usage.

Characteristic	Fitted <2 years old(n = 12)				Fitted ≧2 years old(n = 25)			
Continuous usage	Users		Non-users		Users		Non-users	
	12		0		17		8	
Level of amputation	TR	WD	TC	UR	TR	WD	TC	UR
	7	2	3	0	8(4)*	5(2)	10(2)	2
Situation for usage	Various		At home		Various		At home	
	6		6		9		8	
Rate of discontinuation	N/A				32.0%			

TR: tranradial, WD: wrist disarticulation, TC: transcarpal, UR: ulnar ray N/A: not applicable

*: The number in ( ) indicates the number of Non-users

## Discussion

There is a common understanding that the earlier a child is fitted for a powered prosthesis, the better the result will tend to be [5,6,8,10]. According to reports from various researchers, the rate of discontinuation gets higher when the age at the time of the first prosthesis fitting is two years old or later [4,6,8,9,11,12,13]. Hubbard et al. reported that the rate of discontinuation for children who were initially fitted before two years of age was only 17%, while it was 48% for those who were initially fitted when they were between the ages of two and six years old [6]. Datta et al. reported that the rate of discontinuation for children whose age at the time of the initial powered prosthesis fitting was 20.6 months was 27.3% [12]. Scotland et al. presented their finding that 50% of children who were initially fitted after the age of two years abandoned their prostheses compared with only 22% of children who had been fitted before the age of two years [4]. In our research, 25 children were initially fitted with a powered prosthesis after the age of two years, and other 12 children before two years. However, their discontinuation rate was 21.6% in all cases, and 32.0% in initially fitted after the age of two years cases, which is not worse than those rates reported in past studies. Yet, we have found that children who have their initial powered prosthesis fitting at a relatively later age tend to reject the use of their prosthesis more often. The ages of the 7 children who believed that their powered prosthesis was useless and thus unnecessary were from 9 to 19 years old, respectively. Biddiss et al. reported that rejection rates peaked markedly between the ages of 4 and 10 years old [10]. Scotland et al. argued that termination of the use of a powered prosthesis was more often observed in cases involving children between the ages of six and their early teens [4]. They said that one of the reasons for discontinuation was that the functions of the prosthesis were incapable of fully meeting the needs of those children. The conditions of the seven children who discontinued usage in our research were fully consistent with the findings of the previous reports. In addition, 12 children initially fitted with a powered prosthesis before the age of two years, all of them were between 3 and 12 years old at the latest follow-up, so further careful follow-up as to the rejection of powered prosthesis should be needed. Our result shows that earlier fitting is desirable for continuous use of powered prosthesis. On the other hand, we found no relationship between the age of first fitting and the situation for usage, so introduction of powered prosthesis after 2 years should be considered by judging from children’s needs.

It is believed that another important factor that affects children’s use of powered prosthesis is the level of amputation. According to the opinions of concerned parties, the most desirable level is transradial amputation [4,6,8,9,10,14]. On the other hand, amputations at or below wrist were said to lead to a higher rate of rejection of prosthesis use [4,10,14]. Nonetheless, our research has not found any significant relationship between the amputation level and the rate of discontinuation for powered prosthesis usage. One of the reasons for it may result from our policy that we do not expect children to be all-day user, and we encourage them to find what they want to do with their powered prosthesis. We accept the occasional use of powered prosthesis for particular environment and social activities, even if they wear it less than 1 hour a day. Among the subjects with at or below wrist amputation in our research, there were some children who continued using their prosthesis during such activities as playing violin and practicing flower arrangement. Other previous report that took into consideration the occasional use of prosthesis, Glynn et al. focused on children whose ages at the time of the initial powered prosthesis fitting were between 4 and 15 years old. In their study, they suggested that the discontinuation rate for those with unilateral transradial amputation and those with amputations at or below the wrist was 15.2%, which is not so high [14]. This means that children with transradial amputation and those with amputations at or below wrist can skillfully use their powered prosthesis when they find it useful for fulfilling their needs.

Another reason for our outcome may result from our detailed follow-up. All the children were seen at least every 3 months to catch up their growth and needs of them regarding the powered prosthesis, and we support them to find what they can do and want to do with it. That was more frequent than other reports [4,5]. One of the subjects in our research lived far away from our Center, which made it difficult for us to provide sufficient training and follow-up. Consequently, this child eventually discontinued usage of the prosthesis. We have the opinion that careful detailed follow-up are quite important for introducing powered prosthesis for children. Previous reports have demonstrated that the involvement of the family is also important [1,3]. Various supports from other family members, especially from parents such as putting on the prosthesis, changing batteries, and encouraging their child to use the powered prosthesis is one of the critical factors for prosthetic usage. In our research, all of the families were cooperative with training and using powered prosthesis for their children, and they well understood the importance of home-training and daily maintenance of powered prosthesis. This may result in good acceptance of powered prosthesis in this study.

In addition, we think that another problem is that powered prosthesis is little known in Japan, so the use of prosthesis in schools is not widely accepted yet. Some children in our research, their use of prosthesis was restricted to their home or a particular environment, since they could not get sufficient cooperation from their school, and occasionally they suffered from careless and hurtful comments about their use of powered prosthesis from others (namely, children of the same generation). It goes without saying that sufficient rehabilitation and follow-up are critical for growing children in order to support the continued usage of their prosthesis [6,8,15]. In addition, it is absolutely imperative to secure cooperation from the school. In order to meet these conditions, it is necessary for medical staff, including us, to not only provide training at the rehabilitation facilities, but to also go out to local communities and educate and enlighten residents so as to create and improve environments where children can use prosthesis more easily and comfortably.

## Conclusion

Children with unilateral upper limb deficiencies fitted before 2 years of age tend to accept their powered prosthesis than those fitted after 2 years. Powered prosthesis users could skillfully make use of their prosthesis regardless of the level of amputation. Multidisciprinary team approach, adequate rehabilitation, detailed follow-up and involvement of family members are quite important for introducing powered prosthesis for children.

## References

[1] Hubbard S, Scott R (1992) Hugh MacMillan Rehabilitation Centre pre-school myoelectric experience Myoelectric Prostheses for Infants Fredericton: Institute of Biomedical Engineering.

[2] Mendez M (1985) Evaluation of a myoelectric hand prosthesis for children with a below-elbow absence. Prosthetics and orthotics international 9: 137–140. 4088841 10.3109/03093648509164725

[3] Sörbye R (1989) Upper-extremity amputees: Swedish experiences concerning children Comprehensive Management of the Upper-Limb Amputee: Springer pp. 227–239.

[4] Scotland T, Galway H (1983) A long-term review of children with congenital and acquired upper limb deficiency. Journal of Bone & Joint Surgery, British Volume 65: 346–349. 10.1302/0301-620X.65B3.6841409 6841409

[5] Kuyper M, Breedijk M, Mulders A, Post M, Prevo A (2001) Prosthetic management of children in The Netherlands with upper limb deficiencies. Prosthetics and orthotics international 25: 228–234. 11860097 10.1080/03093640108726606

[6] Hubbard S, Kurtz I, Heim W, Montgomery G (1998) Powered prosthetic intervention in upper extremity deficiency The child with a limb deficiency Rosemont, IL: American Academy of Orthopaedic Surgeons 1998: 417–431.417.

[7] Edelstein J, Berger N (1993) Performance comparison among children fitted with myoelectric and body-powered hands. Archives of physical medicine and rehabilitation 74: 376–380. 8466418

[8] Postema K, Van der Donk V, Van Limbeek J, Rijken RA, Poelma M (1999) Prosthesis rejection in children with a unilateral congenital arm defect. Clinical rehabilitation 13: 243–249. 10392651 10.1177/026921559901300308

[9] Meurs M, Maathuis C, Lucas C, Hadders-Algra M, Van der Sluis C (2006) Prescription of the first prosthesis and later use in children with congenital unilateral upper limb deficiency: A systematic review. Prosthetics and orthotics international 30: 165–173. 16990227 10.1080/03093640600731710

[10] Biddiss E, Chau T (2007) Upper-limb prosthetics: critical factors in device abandonment. American journal of physical medicine & rehabilitation 86: 977–987. 18090439 10.1097/PHM.0b013e3181587f6c

[11] Brooks MB, Shaperman J (1964) Infant prosthetic fitting. A study of the results. The American journal of occupational therapy: official publication of the American Occupational Therapy Association 19: 329–334. 5889678

[12] Datta D, Ibbotson V (1998) Powered prosthetic hands in very young children. Prosthetics and orthotics international 22: 150–154. 9748000 10.3109/03093649809164477

[13] Murray J, Shore B, Trefler E (1972) Prostheses for children with unilateral congenital absence of the hand. J Bone Joint Surg Am 54: 1658–1664. 4653643

[14] Glynn M, Galway H, Hunter G, Sauter W (1986) Management of the upper-limb-deficient child with a powered prosthetic device. Clinical orthopaedics and related research 209: 202–205. 3731596

[15] Routhier F, Vincent C, Morissette M, Desaulniers L (2001) Clinical results of an investigation of paediatric upper limb myoelectric prosthesis fitting at the Quebec Rehabilitation Institute. Prosthetics and orthotics international 25: 119–131. 11573879 10.1080/03093640108726585

